# Hysteria and Certain Allied Conditions

**Published:** 1897-08

**Authors:** 


Hysteria and Certain Allied Conditions. Their Nature and Treatment, with Special Reference to the Application of the Rest Cure, Massage, Electrotherapy, Hypnotism, etc. By George J. Preston, M. D., Professor of Diseases of the Nervous System, College of Physicians and Surgeons, Baltimore ; Visiting Physician to the City Hospital, etc., etc. Small 8vo, pp. 298. Illustrated. Philadelphia: P. Blakiston, Son & Co., 1012 Walnut street. 1897.

This volume consists of a series of eighteen clinical lectures by the scholarly and well-known author at his private hospital at Philadelphia. The different subjects treated are hysteria, recurrent melancholia, irregularly recurrent melancholia, disorders of sleep, choroid movements in an adult male, subjective false sensations of cold, motor ataxia in a child, post-hemiplegic pain, the treatment of sciatica, erythromelalgia, notes on surface temperature, three cases of remarkable spinal anterior curvature, with mental aberration, concerning the history of the discovery of reflex ocular neuroses and the extent to which these reflexes obtain, wrong reference of sensations of pain, pseudocyesis, hysterical contractures, rotatory movements in the feeble-minded.

The subjects enumerated show what a rich supply of material is treated and especially as the majority of the cases are out of the ordinary run.

